# The German national public health institute’s journey to becoming a Key Climate Actor: The Robert Koch Institute’s strategy development process

**DOI:** 10.1093/eurpub/ckac129.227

**Published:** 2022-10-25

**Authors:** A Taylor

**Affiliations:** Department Epidemiology and Health Monitoring, Robert Koch Institute, Berlin, Germany

## Abstract

The Robert Koch Institute (RKI) is Germany's national public health institute. It has been working for a number of years on One Health topics such as zoonotic diseases and antimicrobial resistance, as well as wider areas relevant to climate change such as environmental health. Climate change has, however, only been a specific area of focus since 2019. This was signalled through the creation of a coordination role called One Health Climate Change (OHCC). In addition, a number of projects on climate change have been initiated independently of one another during this time across the RKI. Since summer 2021, RKI has been more active in its strategic development and set up a climate change and health working group of over 50 members of staff representing all departments. This offers a hub structure for pulling the relevant work and activities together under one umbrella, thereby promoting exchange, cooperation and cohesion across RKI. The RKI is currently at an early and dynamic phase in its journey as a key Climate Actor. It is developing a strategy on the topic, guided by the “IANPHI Roadmap for Action on Health and Climate Change” in order to further strengthen its contribution in this area. This presentation will share how RKI has developed its strategy and its action plan for implementation, as well as some of the successes and challenges in their development.

The presentation aims to:

• promote shared learning between national public health institutes on climate change and health strategy development;

• provide an opportunity for shaping future planning at RKI through audience input;

• stimulate thinking on how national public health institutes can remain in exchange with key stakeholder groups and open to learning and adaptation of their strategies in order to be effective Key Climate Actors over the long term.

